# Substance use in childhood and adolescence and its associations with quality of life and behavioral strengths and difficulties

**DOI:** 10.1186/s12889-022-12586-2

**Published:** 2022-02-10

**Authors:** Wiebke Frobel, Nico Grafe, Christof Meigen, Mandy Vogel, Andreas Hiemisch, Wieland Kiess, Tanja Poulain

**Affiliations:** 1 LIFE Leipzig Research Center for Civilization Diseases, Leipzig University, Philipp-Rosenthal-Strasse 27, 04103 Leipzig, Germany; 2 Department of Women and Child Health, University Hospital for Children and Adolescents and Center for Pediatric Research, Leipzig University, Liebigstrasse 20a, 04103 Leipzig, Germany

**Keywords:** Substance use, Alcohol, Smoking, Cannabis, Childhood, Adolescence, Quality of life, Behavioral strength and difficulties

## Abstract

**Background:**

Substance use in childhood and adolescence continues to be a current health concern. The aims of the present study were to identify trends in the use of alcohol, cigarettes, and cannabis in children and adolescents in the last 10 years and to assess associations between substance use and quality of life and behavioral strengths and difficulties.

**Methods:**

Substance use was examined in 1829 9- to 18-year-old German children and adolescents participating in the LIFE Child cohort study between 2011 and 2020. Quality of life was investigated using the KIDSCREEN-27 questionnaire. The Strength and Difficulties Questionnaire was used to assess behavioral strengths and difficulties. Associations were assessed using linear regression analyses. All effects were adjusted for age, gender, and family socio-economic status.

**Results:**

38.44% of participants reported drinking alcohol at least sometimes. Smoking (6.23%) and the use of cannabis (3.94%) were less frequent. While we observed no significant changes in smoking between 2011 and 2021, the consumption of cannabis and the frequent consumption of alcohol has increased in this time period. Cigarette and cannabis use were associated with additional symptoms of hyperactivity/inattention and reduced prosocial behavior. For all three substances, usage was associated with more conduct problems. We also found significant associations between substance use and a lower quality of life in the areas of physical wellbeing, psychological wellbeing, parent relation and autonomy, and school environment. One noteworthy finding was that cigarette consumption and frequent alcohol use were associated with higher quality of life in terms of social support/peer group relations. Some significant interactions between substance use and child age indicated that associations between substance use and quality of life or behavioral difficulties were stronger in younger than in older children.

**Conclusions:**

The results show that quality of life and behavioral difficulties are associated with substance use and should be considered when developing or implementing preventive measures to counter substance use. Furthermore, the findings indicate that substance use can be accompanied by improved peer relations. Therefore, the influence of peers, especially of peers who use these substances, should not be underestimated.

**Supplementary Information:**

The online version contains supplementary material available at 10.1186/s12889-022-12586-2.

## Introduction

The consumption of cigarettes, alcohol or other drugs can trigger the causes or the direct onset of various diseases. In Germany, nicotine and alcohol consumption are among the top 5 avoidable risk factors for illness and death [1]. In most cases, the first contact an individual has with these substances is during childhood and adolescence [2]. During these life stages, the use of substances is particularly critical in terms of developing addictions [2, 3].

In recent years, the consumption of alcohol and cigarettes in Germany has decreased significantly [2]. Still, a large proportion of young people consumes these substances. According to a recent report of the “Health Behaviour in School-aged Children – A WHO Cross National Survey (HBSC)”, the prevalence of smoking in 11- to 15-year-old German boys and girls is about 14% [4]. With regard to alcohol consumption, about 9% of 12- to 17-year-old children in Germany consume alcohol regularly [2], while 51% of children and adolescents report having consumed alcohol at least once in their life [5]. In another report from the HBSC-Study, 24% of girls and 23% of boys reported having consumed alcohol at least once in the last 30 days [6]. In contrast to the consumption of cigarettes and alcohol, cannabis use has risen sharply in recent years [2]. In one study, 4% of young people reported having used cannabis in the previous 30 days, and 2% of 12- to 17-year-olds reported using it regularly [2]. The current Covid-19 pandemic is also leading to an increase in cannabis use among children and adolescents [7].

Several studies investigated associations between substance use in children and adolescents and sociodemographic parameters. Regarding gender differences, girls were reported to be more likely to drink risky amounts of alcohol while boys were more likely to indulge in regular binge drinking (at least 6 drinks or more on one occasion) [5]. Regarding smoking and cannabis use, previous studies revealed that regular use was higher in boys than in girls [2, 4]. With respect to age differences, all three substances (alcohol, cigarettes, cannabis) were shown to be used more frequently with increasing age [2, 4–6]. Evidence about associations between socio-economic status (SES) and substance use in childhood and adolescence is conflicting and inconclusive. A number of studies have found an association between lower SES and higher alcohol consumption [8, 9] and cannabis use [8, 10], however, the results of other studies suggest the opposite [6, 11–13]. Only the use of cigarettes has been consistently correlated with lower SES [10, 11, 14, 15].

The existing research literature links cannabis, alcohol, and cigarette use to several behavioral and psychological disorders in adolescence and young adulthood. Menezes et al. and Dimitrios et al. identified associations between nicotine use and, respectively, depression, somatization, animosity, paranoia, and other behavioral problems [16, 17]. Another study illustrates that smoking in childhood and adolescence can lead to early withdrawal symptoms. In that study, 22% showed the first signs of addiction after only 4 weeks of occasional smoking [18]. Cannabis use has been associated with a higher risk of psychosis [19], bipolar disorder [20], depression, and suicide [21]. Alcohol use has been found to contribute to conduct problems, especially in individuals with depression [22, 23]. Attention Deficit Hyperactivity Disorder has also been reported to be associated with an increased likelihood for alcohol and cigarette use in childhood and adolescence [24, 25]. Importantly, previous research suggests that children who start using substances at an early age are more likely to exhibit behavioral problems than children who start later [26]. These findings show that substance use and behavioral problems can be inter-related, especially in younger children.

Substance use has also been shown to be associated with lower quality of life in children and adolescents [27–30]. This could be caused by reactions of the body, such as symptoms of addiction [18], due to the use of drugs. Further research would be needed to clarify possible mechanisms.

Because substance use in childhood and adolescence can change rapidly, it is important to continually show up-to-date data on current use and associations with well-being and behavioral difficulties. With this in mind, the present study aimed to examine trends in substance use in children and adolescents in the last 10 years. Another aim was to reevaluate the associations between substance use and age, gender, SES, quality of life and behavioral strengths and difficulties in children and adolescents, with a specific focus on possible differences in strengths of associations depending on child age (childhood, early adolescence, late adolescence). This way, the possible targets for prevention and cessation programs can also be adapted to current consumption behavior.

Based on previous studies, we hypothesized that substance use is higher in older children and children with lower SES. Furthermore, we expected that substance use is associated with lower quality of life and more behavioral difficulties, especially in younger children.

## Methods

### Study design and participants

The data for the following project were collected within the framework of the LIFE Child Study in Leipzig, Germany. This prospective cohort study was initiated in 2011 and aims to examine healthy child development with particular interest in the development of lifestyle diseases. Children and adolescents up to age 16 are recruited by advertising at different institutions (e.g., hospitals, schools) and word of mouth. Recruited participants are encouraged to participate in annual follow-up visits. The study program includes various assessments, including medical examinations, interviews, tests, and questionnaires [31, 32]. The study was designed in accordance with the Declaration of Helsinki and its later amendments [33]. It was approved by the Ethics Committee of the Medical Faculty of Leipzig University (Reg. No. 264/10-ek). Informed written consent is obtained from the participants’ parents, for all children, and from the young people themselves if they are aged 12 or above.

**Fig. 1:**
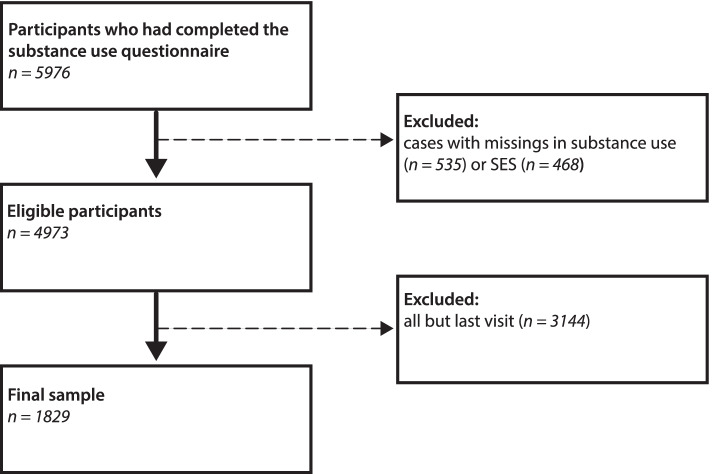
Description of exclusion criteria and the final sample

For the present cross-sectional study, data were collected between 2011 and 2020. The initial survey sample included 5976 data points of 2055 children. In a first step, all cases with missing information about SES (*n* = 468) or substance use (*n* = 535) were excluded (see Fig. 1). In the case of multiple visits per child, only the last visit was included, resulting in a loss of 3144 data points (see Fig. 1). We decided for the last visit, as this allowed for more current data and a more equal age distribution. Including multiple visits from single children would have biased the results. The final sample consisted of 1829 children and adolescents aged between 9.5 and 18.5 years (mean age 13.96 years, 912 boys and 917 girls). Given the broad age range, we distinguished three age groups: childhood (9.5- to 12-year-olds, *n* = 704), early adolescence (13- to 15-year-olds, *n* = 681) and late adolescence (16- to 18.5-year-olds, *n* = 444).

### Assessments

#### Substance use

Substance use (cigarettes, alcohol, and cannabis) was reported by the children and adolescents themselves using a questionnaire. This questionnaire was based on the HBSC-Study though it incorporated several study-specific adjustments [34].

The questions on the frequency of current consumption of cigarettes, alcohol, and cannabis were dichotomized to allow participants to indicate whether they currently use the different substances (yes/no). For alcohol and cigarette use, additional dichotomous (yes/no) variables were used to ascertain whether participants currently consumed these substances frequently, i.e., at least once per week. The questions and the transformation rules are presented in Additional file 1.

#### Socio-economic status

To measure SES, parents were asked to report their monthly net income, their professional activity, and their education. These three components were combined to provide a composite SES score adapted to the Winkler Index, an index used in a German nationwide survey on child health [35] that ranges from 3 points (lowest SES) to 21 points (highest SES) [36]. This score was used as covariate in the analyses. For descriptive purposes, SES was categorized as low, middle, or high, based on cut-offs obtained from a representative German sample [36].

#### Quality of life (Kidscreen-27)

The participants’ quality of life was assessed using the self-report version of the KIDSCREEN-27 questionnaire [37]. The Kidscreen-27 questionnaire was designed to assess quality of life and has been verified in terms of validity and reliability. It has been used in several research projects [37, 38]. The questionnaire assesses the following dimensions of quality of life: physical wellbeing (5 items, Cronbach’s alpha = 0.82), psychological wellbeing (7 items, Cronbach’s alpha = 0.87), parent relations and autonomy (7 items, Cronbach’s alpha = 0.79), social support and peers (4 items, Cronbach’s alpha = 0.86), and school environment (4 items, Cronbach’s alpha = 0.82) [37]. Each of the 27 items is rated on a five-point Likert scale. For each dimension of quality of life, the values of the single items were summed, and the sum scores were transformed to produce age- and gender-specific T-values using reference values from a large reference population [37].

#### Behavioral strengths and difficulties (SDQ)

The behavioral strengths and difficulties of the children and adolescents were assessed using the self-report version of the German version of the Strength and Difficulties Questionnaire (SDQ) [39]. The SDQ comprises 25 items, subdivided into five scales: hyperactivity/inattention (5 items, Cronbach’s alpha = 0.71), emotional symptoms (5 items, Cronbach’s alpha = 0.7), conduct problems (5 items, Cronbach’s alpha = 0.5), peer problems (5 items, Cronbach’s alpha = 0.59), and prosocial behavior (5 items, Cronbach’s alpha = 0.71) [39, 40]. Each item is rated on a three-point Likert scale. For each scale, the values of the single items were summed to sum scores (range 1–10), with higher scores representing greater behavioral difficulties or strengths. Behavioral strength is represented by the prosocial behavior scale; the other scales represent behavioral difficulties [39].

### Statistical analysis

Analyses and visualizations were carried out using R [41]. Descriptive statistics are given as mean and standard deviation (sd) for continuous variables and as count and percentages for categorical variables. To assess the associations between substance use and age, gender, SES, and time of assessment, we applied multiple logistic regression analyses. The five substance use variables were included as dependent variables and age, sex, SES (as continuous measure), and year (2011–2020) were included as independent variables.

Linear regression analyses were applied to explore the associations between quality of life and behavioral difficulties (as dependent variables) and substance use (as independent variables). Analyses were adjusted for sex, age, and SES. Unadjusted associations are presented in Additional file 2. In order to check whether or not associations differed between age groups (childhood versus early adolescence versus late adolescence), each model was checked for interactions between age group and the substance use variable. The age group older adolescence was set as reference. In the case of smoking and frequent smoking, only early and late adolescence could be compared, as child participants did not report smoking at all. An interaction was only reported if the interaction term reached significance and did not cause multicollinearity (variance inflation factor < 5).

To account for multiple testing, *p*-values were adjusted using the False Discovery Rate method [42]. Effects were reported as differences (linear regression) and odds ratios (logistic regressions) including 95% intervals. Associations with a p-value < 0.05 were considered significant.

## Results

### Descriptive statistics

The study sample comprised 1829 children (mean age = 13.96, sd = 2.47, 917 (50.14%) girls). Most of the participants came from middle (*n* = 955, 52%) or high (*n* = 673, 37%) SES families, and 11% (*n* = 201) came from families with low SES [43].

Around 6% of the participants reported current cigarette use. In contrast, nearly 40% of the children and adolescents reported current alcohol use, while about 4% reported current cannabis use (see Table 1). Alcohol use was therefore more frequent than cigarette and cannabis use. Table 1 provides an overview of the study population in terms of the different scales of the KIDSCREEN-27 and SDQ.

**Table 1:** Overview of substance use, behavioral strengths and difficulties, and quality of life (*n* = 1829)

**Substance use**		
Cigarette use	n (%)	114 (6.23%)
Frequent cigarette use	n (%)	81 (4.43%)
Alcohol use	n (%)	703 (38.44%)
Frequent alcohol use	n (%)	96 (5.25%)
Cannabis use	n (%)	72 (3.94%)
**Behavioral strengths and difficulties**		
Prosocial behavior	mean (sd)	7.78 (1.86)
Hyperactivity/inattention	mean (sd)	3.60 (2.18)
Emotional symptoms	mean (sd)	2.43 (2.13)
Conduct problems	mean (sd)	1.69 (1.42)
Peer problems	mean (sd)	2.21 (1.75)
**Quality of life**		
Physical wellbeing	mean (sd)	50.45 (9.77 )
Psychological wellbeing	mean (sd)	50.53 (10.06 )
Parent relations and autonomy	mean (sd)	53.79 (9.97)
Social support & peers	mean (sd)	51.76 (10.56)
School environment	mean (sd)	52.39 (9.55)

Current use: Child currently consumes cigarettes, alcohol, or cannabis at least once per month (Additional file 1); frequent use: Child currently consumes cigarettes or alcohol at least once per week (Additional file 1)

Concerning behavioral difficulties, the average scores were highest for hyperactivity/inattention (mean = 3.60, sd = 2.18) and lowest for conduct problems (mean = 1.69, sd = 1.42). Quality of life was highest in relation to parent relations (mean = 53.79, sd = 9.97), and lowest in terms of physical  wellbeing (mean = 50.45 , sd = 9.77 ).

**Fig. 2:**
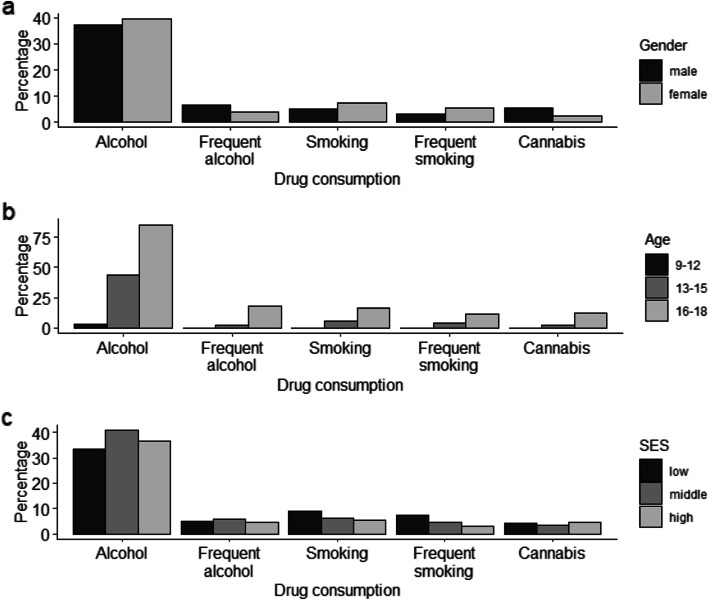
Percentage of substance use depending on gender (**a**), age (**b**), and SES (**c**)

As expected, the likelihood of substance use and frequent substance use increased with increasing age (see Table 2). For example, approximately 4% of the 9- to 12-year-old participants currently consumed alcohol, compared to nearly 44% of the 13- to 15-year-olds and 85% of the 16-to 18-year-olds. This is illustrated in Fig. 2. Cannabis use and frequent alcohol use were less frequent in girls (2 and 4%, respectively) than boys (5 and 7%, respectively, see Table 2 and Fig. 2). Regarding SES, we observed that alcohol use increased with increasing SES (see Table 2 and Fig. 2).

**Table 2:** Associations between substance use and gender, age, SES, and time

Sample *n* = 1829		Current cigarette use^a^	Frequent cigarette use^a^	Current alcohol use^a^	Frequent alcohol use^a^	Current cannabis use^a^
Gender (female)	OR	1.39	1.59	0.93	0.45	0.35
	CI	0.93–2.09	0.99–2.55	0.72–1.21	0.28–0.70	0.20–0.59
	*p*	0.15	0.08	0.70	0.001 **	< 0.001 ***
SES	OR	0.97	0.94	1.05	1.00	1.03
	CI	0.92–1.02	0.88–1.00	1.01–1.09	0.94–1.06	0.96–1.11
	*p*	0.31	0.07	0.02 *	0.99	0.46
Age	OR	1.79	1.72	2.51	2.22	2.15
	CI	1.59–2.00	1.51–1.95	2.31–2.72	1.90–2.59	1.81–2.55
	*p*	< 0.001 ***	< 0.001 ***	< 0.001 ***	< 0.001 ***	< 0.001 ***
Time (in years)	OR	1.08	1.0	0.97	1.14	1.31
	CI	0.99–1.17	0.92–1.1	0.92–1.01	1.02–1.26	1.14–1.5
	*p*	0.08	0.89	0.17	0.01 *	< 0.001 ***

All associations are adjusted for gender, age, and SES

Current use: Child currently consumes cigarettes, alcohol, or cannabis at least once per month (Additional file 1); frequent use: Child currently consumes cigarettes or alcohol at least once per week (Additional file 1)

*CI* Confidence interval, *OR* Odds ratio

*** *p* < .001; ** *p* < .01; * *p* < .05

^a^Reference = no substance use

Looking at time trends in substance use since 2011, the analyses revealed that frequent alcohol use and cannabis use have increased significantly, while the other types of substance use have not changed (see Table 2).

### Associations between substance use and quality of life

Current cigarette use was negatively associated with scores on the KIDSCREEN scales physical wellbeing (beta = − 2.90, *p* = 0.002), psychological wellbeing (beta = −2.92, *p* = 0.003), parent relations (beta = − 2.90, *p* = 0.006), and school environment (beta = − 2.68, *p* = 0.009). Similar associations were observed  for frequent cigarette use (beta_physical_ = − 3.08 , *p* = 0.005, beta_psychological_ = −2.86, *p* = 0.01, beta_parents_ = − 3.99, *p* = 0.001, beta_school_ = − 3.01, *p* = 0.01). For example, for children who reported smoking frequently, the scores for physical wellbeing, psychological wellbeing, parent relations, and school environment were 47.50 45.35 (physical), 47.79 (psychological), 50.26 (parent), and 49.78 (school) points, compared with 50.58  (physical), 50.66 (psychological), 54.24 (parent), and 52.79 (school) points for children who did not report frequent smoking. Interestingly, we observed positive associations between cigarette consumption and social support (beta_current_ = 3.54, *p* = 0.002, beta_frequent_ = 2.97, *p* = 0.03).

Associations with cannabis consumption showed the same pattern. However, only associations with psychological wellbeing, parent relations, and school environment were statistically significant (beta_psychological_ = −3.87, *p* = 0.002, beta_parents_ = − 3.19, *p* = 0.02, beta_school_ = − 4.72, *p* = < 0.001). For children who consume cannabis, the scores for psychological wellbeing, parent relations, and school environment were 46.81, 51.00, and 48.11 points, compared with 50.68, 54.19, and 52.84 points for children who did not report cannabis use. Regarding psychological wellbeing, a significant interaction with age group (*p* = 0.047) showed that the association with cannabis use was significantly stronger in childhood (beta = -23.35) than in late adolescence (beta = -4.09). For quality of life regarding social support, a similar pattern was observed (*p* = 0.038). While there was a weak positive association with cannabis use in late adolescence (beta = 2.56), a strong negative association was observed in early childhood (beta = -19.63). 

Regarding alcohol, there was a negative relationship between both (current and frequent) consumption variables and psychological wellbeing and the school environment score, only reaching significance for current alcohol use (beta_psychological_ = −1.70, *p* = 0.008, beta_school_  = − 1.78, *p* = 0.007). For frequent alcohol use, we found only a positive associations with social support (beta = 2.55, *p* = 0.048). Detailed results including 95% confidence intervals are shown in Table 3.

**Table 3:** Associations between substance use and quality of life (Kidscreen-27)

Sample *n* = 1821		Physical wellbeing	Psychological wellbeing	Parent relations and autonomy	Social support and peers	School environment
Current cigarette use	b	−2.90	−2.92	−2.90	3.54	−2.68
	CI	− 4.74 – –1.07	−4.83 – – 1.01	− 4.83 – − 0.96	1.43–5.64	−4.55 – − 0.81
	*p*	0.002 **	0.003 **	0.006 **	0.002 **	0.009 **
Frequent cigarette use	b	− 3.08	−2.86	−3.99	2.97	−3.01
	CI	– 5.21– – 0.95	−5.08− − 0.64	−6.22 – − 1.75	0.53–5.41	−5.18 – − 0.84
	*p*	0.005 **	0.01 *	0.001 **	0.03 *	0.01 *
Current alcohol use	b	− 0.21 − 0.45	−1.70	−1.26	0.19	−1.78
	CI	− 1.64 − 0.73	−2.93 − − 0.47	−2.51 – − 0.02	− 1.17 – 1.55	− 2.98 – − 0.57
	*p*	0.46	0.008 **	0.07	0.83	0.007 **
Frequent alcohol use	b	0.48	–1.19	0.65	2.55	−1.79
	CI	−1.53 - 2.5	–3.30 – 0.90	−1.47 – 2.77	0.24 – 4.85	−3.84 – 0.26
	*p*	0.64	0.28	0.64	0.048 *	0.12
Current cannabis use	b	−1.35	–3.87	−3.19	2.23	−4.72
	CI	−3.63 − 0.93	−6.23 − −1.5	−5.58 – − 0.80	−0.38 – 4.84	−7.04 – − 2.41
	*p*	0.26	0.002 ** ^a^	0.02 *	0.13 ^a^	< 0.001 ***

All associations are adjusted for gender, age, and SES

Current use: Child currently consumes cigarettes, alcohol, or cannabis at least once per month (Additional file 1); frequent use: Child currently consumes cigarettes or alcohol at least once per week (Additional file 1)

*CI* Confidence interval, *OR* Odds ratio

*** *p* < .001; ** *p* < .01; * *p* < .05

^a^Significant interaction with age indicated stronger (negative) associations in childhood than in late adolescence

### Associations between substance use and behavioral strengths and difficulties

Cigarette consumption was negatively associated with prosocial behavior (beta_current_ = − 0.82, *p* = < 0.001; beta_frequent_ = − 0.84, *p* = 0.001). For children who reported smoking frequently, the score for prosocial behavior was calculated as 6.98 points, compared to 7.81 points for children who did not report frequent smoking. The analyses also revealed significant positive associations between cigarette consumption and hyperactivity/inattention (beta_current_ = 0.85, *p* = 0.001; beta_frequent_ = 0.86, *p* = 0.005) and conduct problems (beta_current_ = 0.95, *p* = < 0.001; beta_frequent_ = 1.11, *p* = < 0.001). As indicated by significant interactions with age group (*p* = 0.005 and 0.040, respectively), the associations between cigarette consumption and conduct problems were stronger in early adolescence (beta_current_ = 1.36, beta_frequent_ = 1.45) than in late adolescence (beta_current_ = 0.46, beta_frequent_ = 0.69).

For cannabis consumption, we observed the same pattern of association as for smoking (hyperactivity: beta = 0.76, *p* = 0.04; conduct problems: beta = 0.94, *p* = < 0.001; prosocial behavior: beta = − 0.85, *p* = 0.007). For example, the score for conduct problems was 2.60 points for children who reported cannabis use and 1.66 points for children reporting no cannabis use. A significant interaction with age group (*p* <  0.001) indicated that the association with prosocial behavior was significantly stronger in childhood (beta = − 7.10) than in late adolescence (beta = − 0.68).

Regarding alcohol, only the association between frequent alcohol use and conduct problems reached statistical significance (beta = 0.48, *p* = 0.02). For children who reported frequent alcohol use, the score for conduct problems was calculated as 2.14 points, compared to 1.67 points for children who reported no or infrequent alcohol use. However, for the associations between frequent alcohol use and emotional problems as well as peer relationship problems, the analyses revealed significant interactions with age group (*p* = 0.004 and 0.015, respectively). Both interactions indicated stronger (positive) associations in childhood (beta = 5.40 and 3.79, respectively) than in late adolescence, where associations were even negative, but very weak (beta = − 0.47 and − 0.45, respectively). Detailed results including 95% confidence intervals are shown in Table 4.

**Table 4:** Associations between substance use and behavioral difficulties and strengths (SDQ)

Sample *n* = 1536		Prosocial Behavior	Hyperactivity/Inattention	Emotional symptoms	Conduct problems	Peer problems
Current cigarette use	b	−0.82	0.85	−0.03	0.95 ^a^	0.20
	CI	−1.23 – −0.41	0.37 – 1.34	−0.50 – 0.43	0.64 – 1.27	−0.20 – 0.59
	*p*	< 0.001***	0.001 **	0.91	< 0.001 ***	0.41
Frequent cigarette use	b	−0.84	0.86	0.02	1.11 ^a^	0.01
	CI	−1.31 – −0.36	0.30 – 1.42	−0.51 – 0.56	0.75 – 1.47	−0.44 – 0.47
	*p*	0.001 **	0.005 **	0.94	< 0.001 ***	0.96
Current alcohol use	b	0.02	0.11	0.05	0.19	−0.09
	CI	−0.22 – 0.26	−0.17 – 0.40	− 0.22 – 0.33	0.00 – 0.37	−0.32 – 0.14
	*p*	0.90	0.52	0.76	0.07	0.54
Frequent alcohol use	b	−0.07	0.19	−0.23 ^b^	0.48	−0.16
	CI	−0.54 – 0.41	−0.37 – 0.75	− 0.77 – 0.30	0.11 – 0.84	−0.61 – 0.29
	*p*	0.84	0.59	0.48	0.02 *	0.58
Current cannabis use	b	−0.85	0.76	0.31	0.94	0.06
	CI	−1.43 – −0.28	0.08 – 1.44	−0.34 – 0.96	0.50 – 1.37	−0.49 – 0.62
	*p*	0.007 **	0.04 *	0.43	< 0.001 ***	0.85

All associations are adjusted for gender, age, and SES

Current use: Child currently consumes cigarettes, alcohol, or cannabis at least once per month (Additional file 1); frequent use: Child currently consumes cigarettes or alcohol at least once per week (Additional file 1)

*CI* Confidence interval, *OR* Odds ratio

*** *p* < .001; ** *p* < .01; * *p* < .05.1

^a^Significant interaction with age indicated stronger (negative) associations in early than in late adolescence

^b^Significant interaction with age indicated stronger (negative) associations in childhood than in late adolescence

## Discussion

The present project examined associations between substance use and age, gender, SES, time of assessment, quality of life, and behavioral strengths and difficulties in 9- to 18-year-old German children and adolescents. Of the 1829 surveyed participants, about 6% reported that they currently smoked and nearly 4% reported current cannabis use. These results are comparable to other German studies [2, 5], for example the HBSC-Study, where the reported 30-day-prevalence was about 7% for smoking [4] and around 8% (girls) and 10% (boys) for cannabis use [44]. In our data, the prevalence of current alcohol use was nearly 40%. In comparison, one prior study indicated a 30-day-prevalence for alcohol use of around 23% [6], while another reported regular alcohol use by 9% of participants [2]. As the timeframe in question is different in each of these studies, it is difficult to compare the results directly. Nevertheless, the data indicate that, of the substances under consideration, alcohol is the one that German children and adolescents use most frequently.

### Substance use and socio-economic status, age, gender, and time of assessment

We observed a positive association between current alcohol use and higher SES. Even if this finding contradicts previous studies and our expectations, it is in line with some other studies [6, 11, 13]. It is possible that, compared to other substances, alcohol has less of a negative image and drinking might be perceived as more acceptable in higher SES groups. Other research has indicated that wealthier or more educated parents are more likely to talk positively about the taste or benefits of alcoholic drinks in front of their children [45]. As such, the use of substances by parents, as role models [46], may also provide some explanation for this finding. It is worth noting that, in this analysis, we did not distinguish different types of alcoholic drink. Associations with SES might be different for different beverages. Surprisingly, we did not observe a significant association between lower SES and smoking. This contradicts previous studies [10, 11, 14, 15]. The associations in the present study did point in the expected direction but did not reach significance. One possible explanation for this is that the participants in our study tend to come from families with a higher social status compared to the general population. This could have biased in these results.

As expected, substance use increased with increasing age. This is consistent with other studies [2, 5]. As the children and adolescents get older, they also become more independent and might be more likely to try new things, to follow the example of their family [47] and friends [5], or to be influenced by the various ways alcohol and cigarette use is promoted in the media [48, 49].

Regarding gender, our analyses indicated that boys consume more alcohol and cannabis than girls. These findings coincide with findings of a large German cohort study [2]. A possible explanation is that boys tend to exhibit more risky behavior than girls. The tendency to seek out adventure and excitement (sensation seeking) has also been shown to be higher in boys than girls [50]. In contrast to other studies [2, 4] but in accordance with another German study [5], our analyses did not show significant gender differences regarding smoking. This finding might be explained by changing role models, especially by the increasing gender equality [51]. In the period after the First World War, smoking was considered as a male privilege [52]. Nowadays, stereotypical masculine behavior patterns are increasingly adopted by (young) women as well [53].

Regarding time trends in substance use, the analyses revealed no changes in smoking and current consumption of alcohol. The frequent consumption of alcohol (i.e., at least once per week) and the consumption of cannabis even increased. While the increase in cannabis consumption is in line with other studies [2], the other findings contradict previous studies that suggest a slight decrease in substance use [2, 5]. Our findings show that substance use in children and adolescence remains an important health issue.

### Substance use and quality of life

In line with our hypotheses and previous studies [27, 28], we observed lower quality of life in children and adolescents who use substances, especially in the areas of physical wellbeing, psychological wellbeing, parent relations, and school environment. These findings indicate that the use of substances might primarily affect (or be affected by) physical health, psychological health, and the relationship with adults (teachers and parents), possibly due to drug-use related physical [18, 54, 55] and psychological symptoms^17^, ^19^, ^23^, ^56^ or family and school conflicts. 

In one surprising finding, we observed greater satisfaction with peer relationships in children and adolescents who reported (frequently) consuming cigarettes or alcohol. One possible reason for this finding is that doing something together with friends (e.g., substance use) generates positive feelings, thereby contributing to the satisfaction with peer relationships [57]. Another German study has also shown that children and adolescents are more likely to smoke if their friends smoke [5]. The need to belong or fit in is a fundamental human need [58] and might explain why children and adolescents use substances to increase their popularity within a peer group [59], something that might also lead to improved wellbeing. A recent study indicated that, during the COVID-19 pandemic, adolescents with a self-reported low popularity were concerned about the impact of social distancing on their peer reputation, and that this concern was an influencing factor in substance use in the company of their friends [7]. The findings show how important the effects of peer groups and the social environment are. This might be a basis for prevention programs designed to counteract substance use in childhood and adolescence. 

### Substance use and behavioral strengths and difficulties

Our analysis revealed associations between substance use and externalizing behavioral difficulties (hyperactivity/inattention, conduct problems) and low prosocial behavior. In contrast, we did not find any significant associations between substance use and internalizing behavioral difficulties. Our findings are consistent with the results of other studies [16, 17, 19, 20, 23]. Sensation seeking, a personality trait that, unfortunately, was not assessed in our study, may provide some explanation for these patterns of association. Sensation seeking has been shown to be associated with alcohol problems [60–62], smoking and cannabis use [62], but also with antisocial behavior [63] and other externalizing problems [64, 65]. Therefore, sensation seeking might be a trigger for both substance use and externalizing behavioral difficulties.

### Differences in associations depending on child age

Some of the associations between substance use and behavioral difficulties and quality of life – namely associations between cannabis consumption and psychological wellbeing and prosocial behavior, between smoking and conduct problems, and between frequent consumption of alcohol and emotional and peer relationship problems – were significantly stronger in younger children than in older adolescents. As already suggested by other studies [26], these findings suggest that substance use is especially harmful in childhood or that lower wellbeing and behavioral difficulties in younger children might more easily lead to early substance use.

### Limitations

One limitation of our study is the limited representativeness of the study population, especially regarding SES. In comparison to a representative German sample, the lower SES stratum was slightly under-represented, while the higher stratum was over-represented [43]. Although this was considered during the analysis, the results must, therefore, be viewed with caution. As reported, a higher alcohol use was associated with higher SES. However, since, proportionally, our study population is weighted in the direction of the upper SES stratum, possible biases may be present.

Furthermore, it is not clear whether substance use affects quality of life and behavior, or whether it is the other way around. Also, the data says nothing about individual trajectories of substance use. This would require longitudinal observations. Another limitation is that the data are entirely based on self-reported information provided by the children and adolescents. The veracity of the information provided is not supported by any additional data.

## Conclusion

The results of the present study show that many children and adolescents continue to use the substances discussed here, and that substance use in childhood and adolescence is associated with behavior difficulties and a lower quality of life, especially in younger children. These findings underline the importance of preventive measures to counteract substance use. Furthermore, the influence of young people’s peer groups should not be underestimated, since it is possible that substance use can strengthen or maintain their popularity and wellbeing within a group.

## Supplementary Information

**Additional file 1.** Transformation of the substance use questionnaire. The used questions an d the transformation rules of the final substance variables.

**Additional file 2.** Unadjusted regression coefficients for all associations. Unadjusted regression coefficients for all associations bet w een substa nce use and Kidscreen and SDQ.

## Data Availability

The datasets generated and/or analyzed during the current study are not publicly available due to ethical restrictions. The LIFE Child study is a study collecting potentially sensitive information. Publishing data sets is not covered by the informed consent provided by the study participants. Furthermore, the data protection concept of LIFE requests that all (external as well as internal) researchers interested in accessing data sign a project agreement. Researchers that are interested in accessing and analyzing data collected in the LIFE Child study may contact the data use and access committee (forschungsdaten@medizin.uni-leipzig.de).

## Abbreviations

CI: Confidence interval
HBSC: Health Behaviour in School-aged Children – A WHO Cross National Survey
OR: Odds ratio
SD: Standard deviation
SDQ: The Strength and Difficulties Questionnaire
SES: Socio-economic status
