# Supplementary material for: Substance use in childhood and adolescence and its associations with quality of life and behavioral strengths and difficulties
Source: BMC Public Health. 2022 Feb 10;22:275. doi: 10.1186/s12889-022-12586-2 (PMC8831000; doi:10.1186/s12889-022-12586-2)
Supplement: Supplementary file 1 — Additional file 1. Transformation of the substance use questionnaire. The used questions an d the transformation rules of the final substance variables. [file 12889_2022_12586_MOESM1_ESM.pdf]

Additional file

Article: Substance use in childhood and adolescence and its associations with quality of life and behavioral strengths and difficulties

Authors: Wiebke Frobel, Nico Grafe, Christof Meigen, Mandy Vogel, Andreas Hiemisch, Wieland Kiess, and Tanja Poulain

Journal: BMC Public Health

Transformation of the substance use questionnaire

Questions of the Substance use questionnaire and variables used for further analysis:	Fragen des Drogen-Fragebogens und Variablen, die für die Analyse genutzt wurden:
**Cigarettes:**	**Zigaretten:**
**Question: How often do you smoke?**<br>**Response options:**<br>1 I do not smoke / I quit smoking<br>2 Less than once a week<br>3 At least once a week, but not daily<br>4 Every day<br><br>**Variable Current cigarette use:**<br>Response options 2, 3, or 4<br><br>**Variable Frequent cigarette use:**<br>Response options 3 and 4	**Frage: Wie oft rauchst du?**<br>**Antwortmöglichkeiten:**<br>1 Ich rauche nicht / habe mit dem Rauchen aufgehört<br>2 Weniger als einmal pro Woche<br>3 Mindestens einmal pro Woche, aber nicht täglich<br>4 Jeden Tag<br><br>**Variable zum derzeitiger Zigarettenkonsum:**<br>Antwortmöglichkeiten 2, 3 oder 4<br><br>**Variable zur Häufigkeit des Zigarettenkonsums:**<br>Antwortmöglichkeiten 3 und 4
**Alcohol:**	**Alkohol:**
**Questions: How often do you currently drink?**<br>- Beer?<br>- Wine?<br>- Sparkling wine?<br>- Liqueur?<br>- Spirits (hard liquor)?<br>- Alcoholic mixed drinks?<br>- Shandy (beer mixed with a soft drink)?<br>- Other?<br>**Response options:**<br>1 Never<br>2 Less than once a month<br>3 Every month<br>4 Every week	**Fragen: Wie oft trinkst du derzeit?**<br>- Bier?<br>- Wein?<br>- Sekt?<br>- Likör?<br>- Schnaps<br>- Alkopops?<br>- Biermixgetränke?<br>- Anderes?<br>**Antwortmöglichkeiten:**<br>1 Nie<br>2 Seltener als einmal pro Monat<br>3 Jeden Monat<br>4 Jede Woche

5 Every day<br><br>**Variable current alcohol consumption:**<br>Response options 2, 3, 4, or 5 for at least one of the alcoholic drink types<br><br>**Variable frequent alcohol consumption:**<br>Response options 4 or 5 for at least one of the alcoholic drink types	5 Jeden Tag<br><br>**Variable zum derzeitiger Alkoholkonsum:**<br>Antwortmöglichkeiten 2, 3, 4 oder 5 für mindestens eines der alkoholischen Getränke<br><br>**Variable zur Häufigkeit des Alkoholkonsums:**<br>Antwortmöglichkeit 4 oder 5 für mindestens eines der alkoholischen Getränke
**Cannabis:**	**Cannabis:**
**Question: How often have you consumed cannabis, hashish, or marijuana in the past 30 days?**<br>**Response options:**<br>1 Never<br>2 1 to 2 times, on 1 to 2 days<br>3 3 to 5 times, on 3 to 5 days<br>4 6 to 9 times, on 6 to 9 days<br>5 More than 10 times, on more than 10 days<br><br>**Variable current cannabis consumption**:<br>Response options 2, 3, 4, or 5	**Frage: Wie oft hast du in den letzten 30 Tagen Cannabis, Haschisch oder Marihuana konsumiert?**<br>**Antwortmöglichkeiten:**<br>1 Nie<br>2 1- bis 2-mal / an 1 bis 2 Tagen<br>3 3- bis 5- mal / an 3 bis 5 Tagen<br>4 6- bis 9- mal / an 6 bis 9 Tagen<br>5 Mehr als 10- mal / an mehr als 10 Tagen<br><br>**Variable zum derzeitiger Cannabiskonsum**<br>Antwortmöglichkeit 2, 3, 4 oder 5
