# Supplementary material for: Substance use in childhood and adolescence and its associations with quality of life and behavioral strengths and difficulties
Source: BMC Public Health. 2022 Feb 10;22:275. doi: 10.1186/s12889-022-12586-2 (PMC8831000; doi:10.1186/s12889-022-12586-2)
Supplement: Supplementary file 2 — Additional file 2. Unadjusted regression coefficients for all associations. Unadjusted regression coefficients for all associations bet w een substa nce use and Kidscreen and SDQ. [file 12889_2022_12586_MOESM2_ESM.pdf]

Additional file 2

Article: Substance use in childhood and adolescence and its associations with quality of life and behavioral strengths and difficulties

Authors: Wiebke Frobel, Nico Grafe, Christof Meigen, Mandy Vogel, Andreas Hiemisch, Wieland Kiess, and Tanja Poulain

Journal: BMC Public Health

Unadjusted regression coefficients for all associations:

Vaiable		Current Smoking	Frequent Smoking	Current Alcohol	Frequent Alcohol	Current Cannabis
**Kidscreen**:						
Physical wellbeing	b	-5.46	-5.85	-3.00	-1.61	-2.88
	p	<0.001 ***	<0.001 ***	<0.001 ***	0.12	0.01 *
Psychological wellbeing	b	-5.15	-5.28	-3.32	-2.66	-4.80
	p	<0.001 ***	<0.001 ***	<0.001***	0.01 *	<0.001 ***
Parent relations and	b	-2.58	-3.94	0.03	1.55	-1.71
autonomy	p	0.007 **	<0.001 ***	0.95	0.14	0.16
Social support and peers	b	3.11	2.59	0.009	2.12	1.96
	p	0.002**	0.03*	0.99	0.06	0.13
School Environment	b	-4.53	-4.93	-3.27	-3.78	-6.35
	p	<0.001 ***	<0.001 ***	<0.001 ***	<0.001 ***	<0.001 ***
**SDQ**:						
Prosocial behavior	b	-0.5	-0.55	0.3	0.08	-0.75
	p	0.02 *	0.03 *	0.002 **	0.75	0.01*
Hyperactivity/Inattention	b	0.33	0.39	-0.56	-0.37	0.24
	p	0.19	0.18	<0.001 ***	0.2	0.5
Emotional problems	b	0.3	0.37	0.28	-0.15	0.21
	p	0.21	0.19	0.01 *	0.58	0.54
Conduct problems	b	0.59	0.79	-0.29	0.09	0.58
	p	<0.001 ***	<0.001 ***	<0.001***	0.61	0.01 *
Peer problems	b	0.17	0.02	-0.17	-0.22	-0.07
	p	0.38	0.92	0.06	0.33	0.81

*** p < .001; ** p < .01; * p < .05
